# Author Correction: Hallmarks of a genomically distinct subclass of head and neck cancer

**DOI:** 10.1038/s41467-025-57020-4

**Published:** 2025-02-17

**Authors:** Tara Muijlwijk, Irene H. Nauta, Anabel van der Lee, Kari J. T. Grünewald, Arjen Brink, Sonja H. Ganzevles, Robert J. Baatenburg de Jong, Lilit Atanesyan, Suvi Savola, Mark A. van de Wiel, Laura A. N. Peferoen, Elisabeth Bloemena, Rieneke van de Ven, C. René Leemans, Jos B. Poell, Ruud H. Brakenhoff

**Affiliations:** 1 Amsterdam UMC, location Vrije Universiteit Amsterdam, Otolaryngology / Head and Neck Surgery, Amsterdam, The Netherlands; 2 Cancer Center Amsterdam, Cancer Biology and Immunology, Amsterdam, The Netherlands; 3 Amsterdam Institute for Infection and Immunity, Cancer Immunology, Amsterdam, The Netherlands; 4 Erasmus University Medical Center, Otorhinolaryngology / Head and Neck Surgery, Rotterdam, Netherlands; 5 MRC Holland, Oncogenetics, Amsterdam, The Netherlands; 6 Amsterdam UMC, Epidemiology & Data Science, Amsterdam Public Health Research Institute, Amsterdam, The Netherlands; 7 Amsterdam UMC, location Vrije Universiteit Amsterdam, Pathology, Amsterdam, The Netherlands; 8 Academic Center for Dentistry, Maxillofacial Surgery/ Oral Pathology, Amsterdam, The Netherlands

**Keywords:** Head and neck cancer, Cancer genomics, Tumour heterogeneity, Oral cancer

Correction to: *Nature Communications* 10.1038/s41467-024-53390-3, published online 20 October 2024

In the version of the article initially published, there was a mistake in reported *P* values in the third paragraph of the Results “CNA-quiet oral cavity SCC are clinically distinct with more favorable prognosis” subsection, where in the text now reading “When the analysis was restricted to early-stage lesions, no significant difference was found (*p* = 0.22) for CNA-quiet versus CNA-other. However, the more favorable prognosis of CNA-quiet OCSCC was particularly pronounced in late-stage disease (III–IV, *p* = 0.02),” the values “*p* = 0.22 and “*p* = 0.02 originally read “*p* = 0.21” and “*p* = 0.003.” In the Supplementary information originally published, Supplementary Fig. 13 was an earlier, incorrect version. The text and Supplementary information are updated in the HTML and PDF versions of the article.

## Supplementary information

Updated Supplementary Information

