# Supplementary material for: Hallmarks of a genomically distinct subclass of head and neck cancer
Source: Nat Commun. 2024 Oct 20;15:9060. doi: 10.1038/s41467-024-53390-3 (PMC11491468; doi:10.1038/s41467-024-53390-3)
Supplement: Supplementary file 1 — Supplementary Information [file 41467_2024_53390_MOESM1_ESM.pdf]

-Supplementary Information-

Hallmarks of a Genomically Distinct Subclass of Head and Neck Cancer

Tara Muijlwijk[1,2,3], Irene H. Nauta[1,2], Anabel van der Lee[1,2,3], Kari J.T. Grünewald[1,2], Arjen Brink[1,2], Sonja H. Ganzevles[1,2,3], Robert J. Baatenburg de Jong[4], Lilit Atanesyan[5], Suvi Savola[5], Mark A. van de Wiel[6], Laura A.N. Peferoen[2,7,8], Elisabeth Bloemena[2,7,8], Rieneke van de Ven[1,2,3], C. René Leemans[1,2], Jos B. Poell[1,2] & Ruud H. Brakenhoff[1,2*]*

[1] Amsterdam UMC, location Vrije Universiteit Amsterdam, Otolaryngology / Head and Neck Surgery, Amsterdam, The Netherlands
[2] Cancer Center Amsterdam, Cancer Biology and Immunology, Amsterdam, The Netherlands
[3] Amsterdam Institute for Infection and Immunity, Cancer Immunology, Amsterdam, The Netherlands
[4] Erasmus University Medical Center, Otorhinolaryngology / Head and Neck Surgery, Rotterdam, Netherlands
[5] MRC Holland, Oncogenetics, Amsterdam, The Netherlands
[6] Amsterdam UMC, Epidemiology & Data Science, Amsterdam Public Health Research Institute, Amsterdam, The Netherlands
[7] Amsterdam UMC, location Vrije Universiteit Amsterdam, Pathology, Amsterdam, The Netherlands
[8] Academic Center for Dentistry, Maxillofacial Surgery/ Oral Pathology, Amsterdam, The Netherlands
* shared last authors and to whom all correspondence should be addressed:
j.poell@amsterdamumc.nl, rh.brakenhoff@amsterdamumc.nl

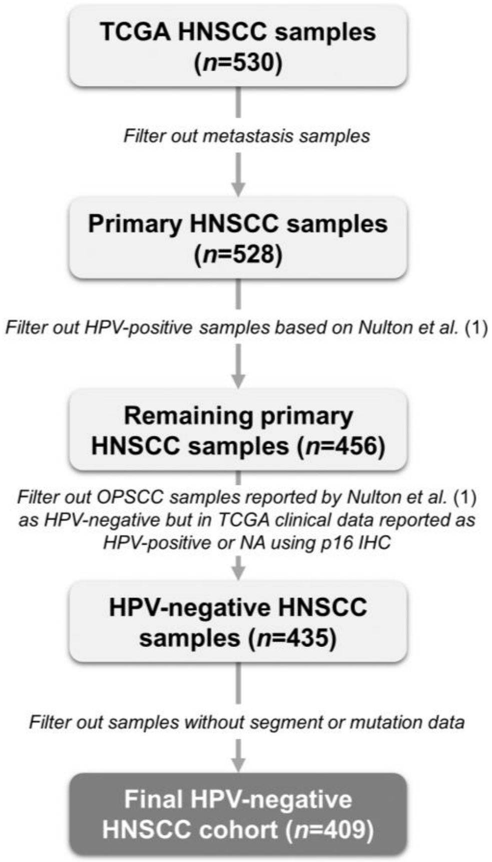

Supplementary Fig. 1 | Filtering of the human papilloma virus-negative head and neck squamous cell carcinoma TCGA. Clinical data of 530 head and neck squamous cell carcinoma (HNSCC) samples from TCGA dataset were downloaded; 528 primary HNSCC samples were left after filtering out two metastasis samples. Human papillomavirus (HPV)-positive samples were filtered out based on detection of viral transcripts, as reported by Nulton *et al.* (1). In addition, oropharynx squamous cell carcinoma (OPSCC) samples reported by Nulton *et al.* (1) as HPV-negative but described as HPV-positive or NA in TCGA dataset using p16 immunohistochemistry (IHC) were filtered as well. From the 435 HPV-negative HNSCC samples, segment and mutation data was unavailable for 26 samples, leaving us with a cohort of 409 HPV-negative HNSCC for further analysis. Source data are provided with this paper.

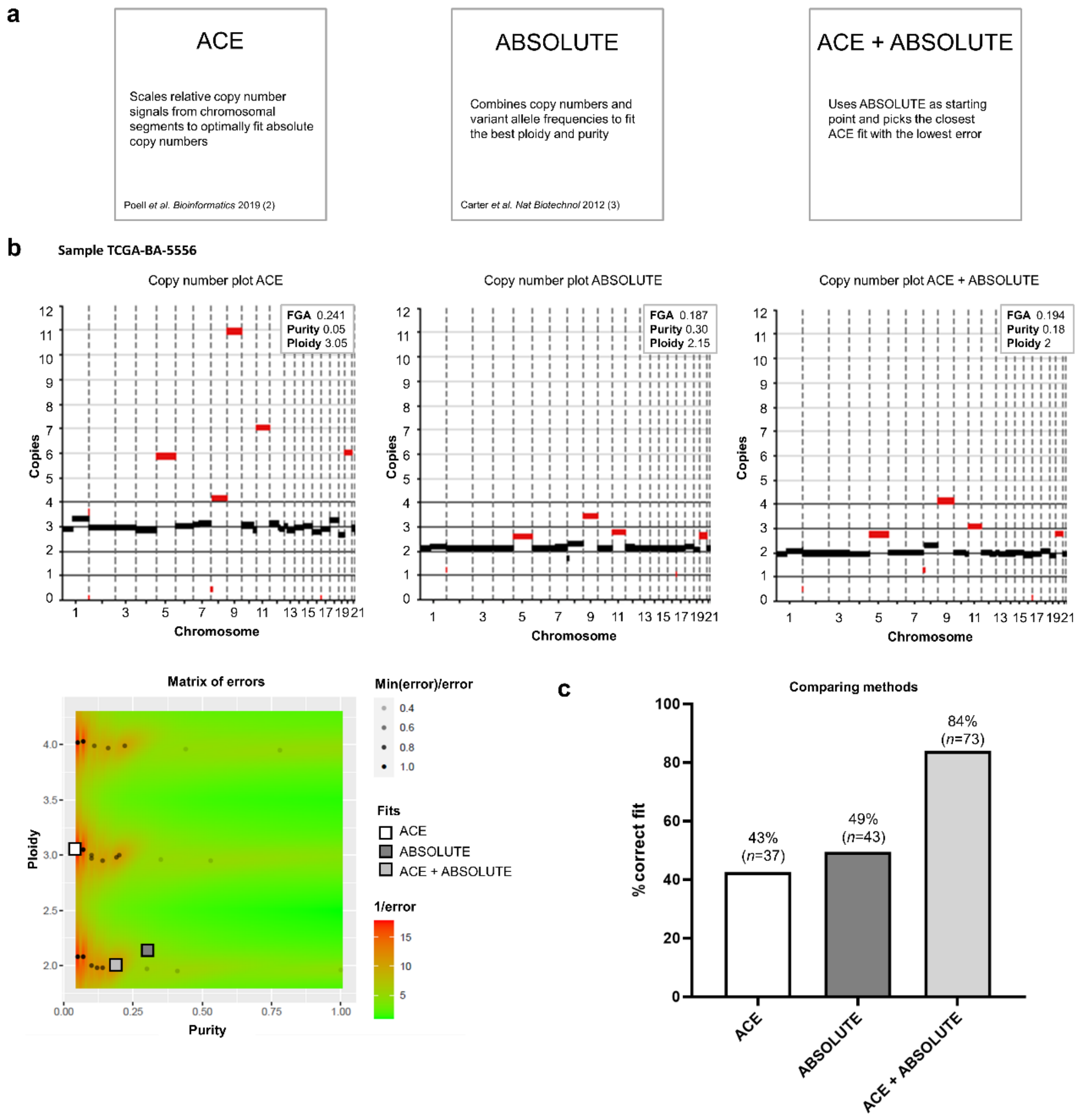

Supplementary Fig. 2 | Evaluating R packages ACE, ABSOLUTE and the combination to infer the purity, ploidy, copy numbers and purity from TCGA human papilloma virus-negative head and neck squamous cell carcinoma cohort. a, R package ACE (Poell *et al.* (2)) scales relative copy number signals from chromosomal segments to optimally fit absolute copy numbers, ABSOLUTE (Carter *et al.* (3)) combines copy numbers and variant allele frequencies to fit the best ploidy and purity, ACE + ABSOLUTE uses ABSOLUTE as starting point and picks the closest ACE fit with the lowest error. **b,** Representative sample of TCGA with copy number plots using ACE (left), ABSOLUTE (middle) and ACE + ABSOLUTE (right) and accompanying matrix of errors. Segments are shown in black and highlighted in red when called. **c,** Result of scoring all TCGA human papilloma virus (HPV)-negative head and neck squamous cell carcinoma (HNSCC) samples with provided FGA < 0.10 (n=87) using ACE, ABSOLUTE and ACE + ABSOLUTE. When fits were equally performing they were both scored as correct.

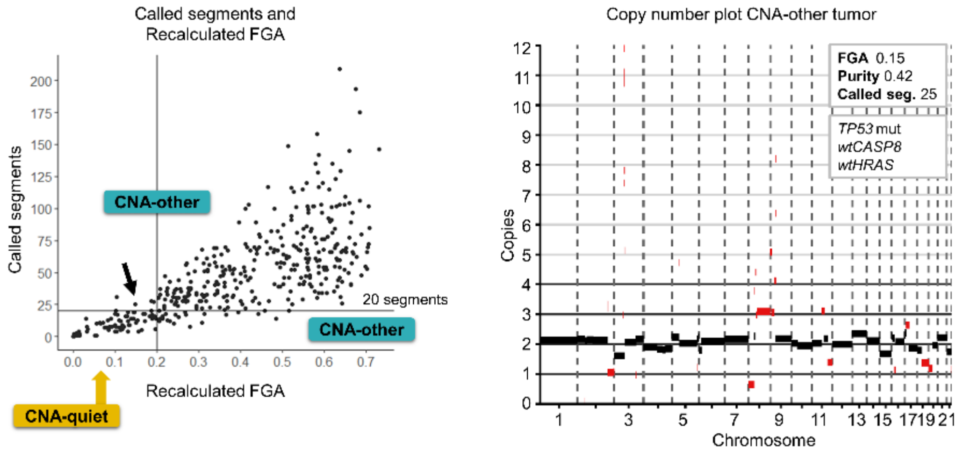

Supplementary Fig. 3 | Copy number plot of copy number alteration-other TCGA tumor. Called segments and recalculated fraction genome altered (FGA) in left panel, as in Fig. 1g. Arrow indicates representative sample with FGA < 0.20 but ≥ 20 called segments. Source data are provided with this paper. Segments are shown in black and highlighted in red when called.

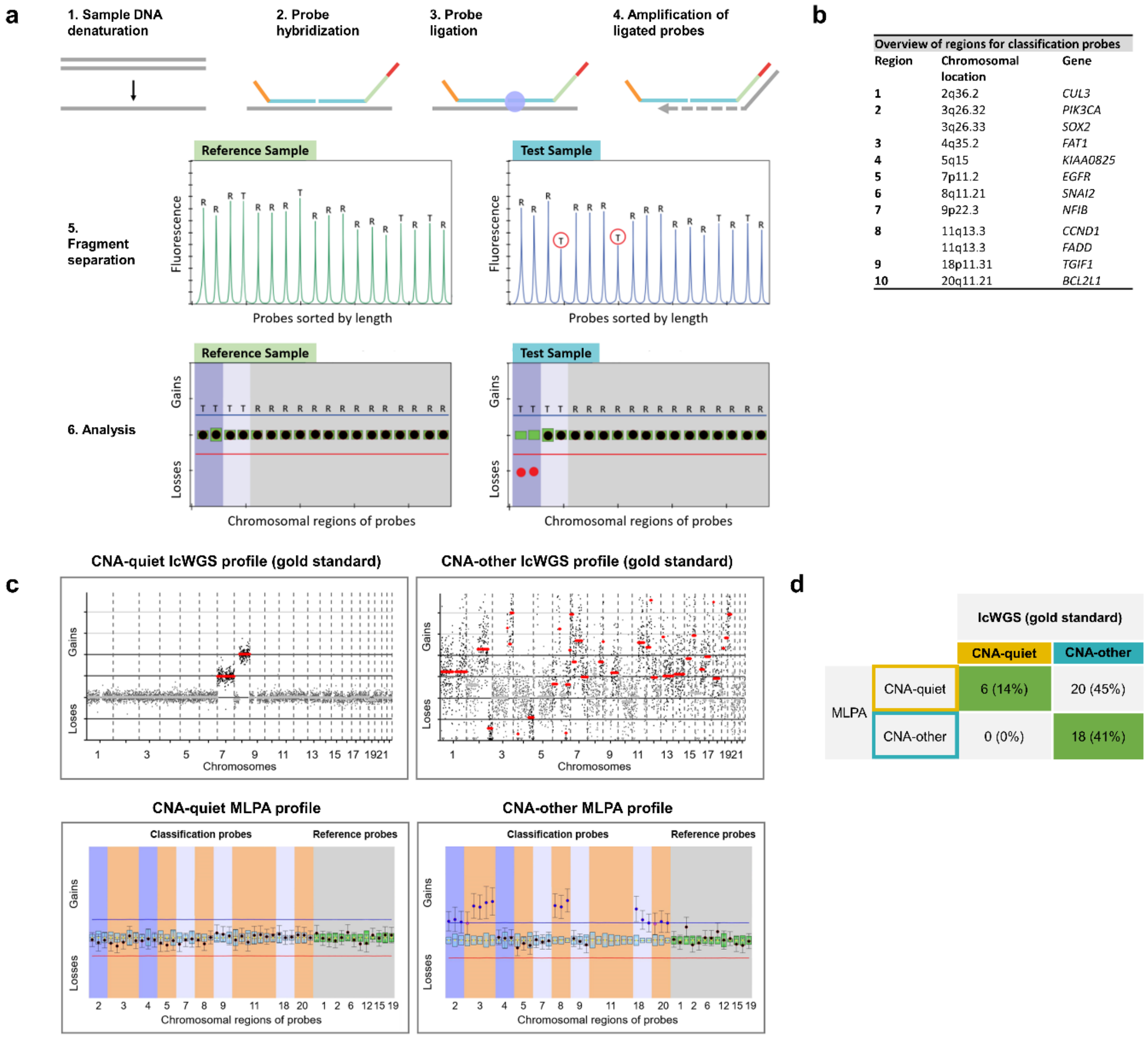

Supplementary Fig. 4 | Pre-screening method multiplex ligation-dependent probe amplification. a, Schematic representation of workflow of multiplex ligation-dependent probe amplification (MLPA) with 1. DNA denaturation, 2. probe hybridization, 3. probe ligation, 4. amplification of ligated probes, 5. fragment separation and 6. data analysis. **b,** Overview of ten most frequently gained and lost regions in human papilloma virus-negative head and neck cancer tumors used for the selection of classification probes. **c,** Representative example of a copy number alteration (CNA)-quiet and CNA-other copy number profile using the gold standard low coverage whole genome sequencing (lcWGS) as well as CNA-quiet and -other MLPA profiles. Segments are shown in black and highlighted in red when called. **d,** Results of a pilot evaluating MLPA as pre-screening method using 44 head and neck squamous cell carcinoma samples with lcWGS as gold standard.

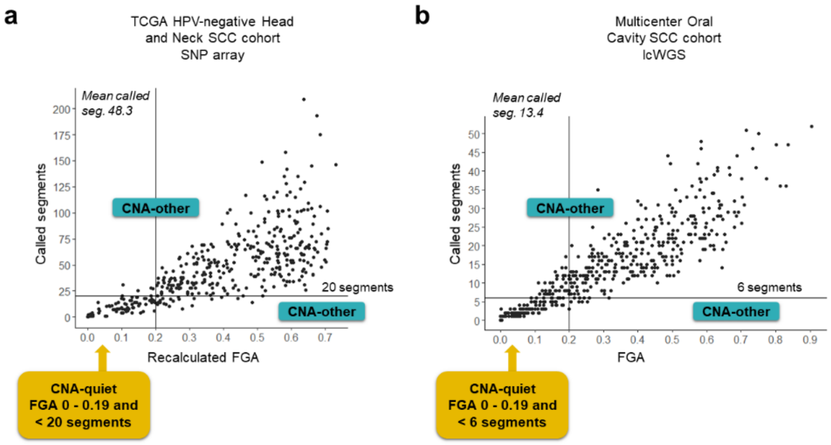

Supplementary Fig. 5 | Cut-off for copy number alteration (CNA)-quiet tumors in TCGA human papillomavirus (HPV)-negative head and neck squamous cell carcinoma (SCC) cohort and multicenter oral cavity SCC cohort. Called segments (y-axis) and fraction genome altered (FGA, x-axis) were used to classify CNA-quiet tumors in the **a,** TCGA cohort and **b,** Multicenter cohort. To translate cut-off for CNA-quiet tumors from TCGA cohort, where data was obtained by single nucleotide polymorphisms (SNP) array, to the multicenter cohort, where data was obtained by low coverage whole genome sequencing (lcWGS), average called segments were calculated and the ratio was used to calculate the segment cut-off for the multicenter oral cavity SCC cohort.

a

Representative summary figures CNA-quiet oral cavity SCCs

Sample 502

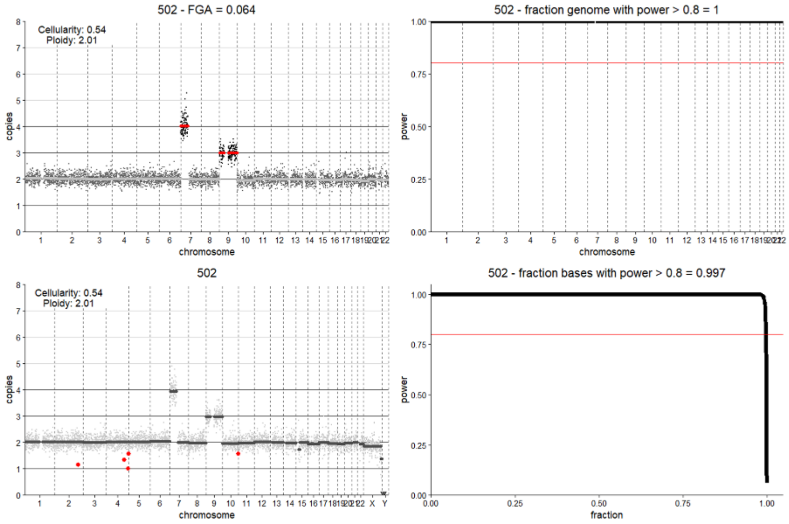

CHROM	POS	GENE	REF	ALT	DP	AD	VAF	change	ClinVar	Copynumbers	Mutant copies
chr11	534285	HRAS	C	T	127	54	0.425	p.G13D	Pathogenic	1.990	1.571
chr2	202149770	CASP8	G	A	156	48	0.308	p.C345Y		2.022	1.146
chr4	153247175	FBXW7	T	C	126	45	0.357	p.R543G		2.015	1.328
chr4	187535437	FAT1	G	C	141	38	0.270	p.S3046C		2.015	1.002
chr4	187539951	FAT1	G	A	123	33	0.268	p.R2597*		2.015	0.998
chr5	1295228	TERT	G	A	64	27	0.422	FIVE_PRIME_FLANK		2.015	1.569

Sample 699

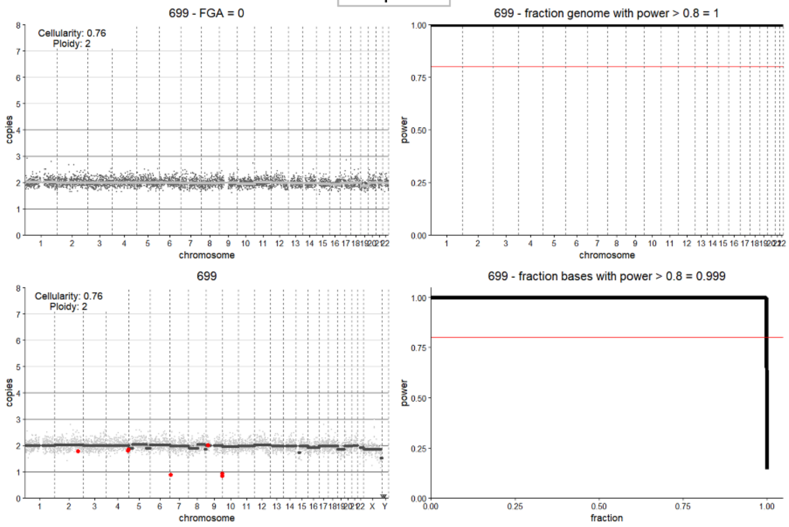

CHROM	POS	GENE	REF	ALT	DP	AD	VAF	change	ClinVar	Copynumbers	Mutant copies
chr2	202149668	CASP8	T	G	304	204	0.671	p.I311S		2.017	1.777
chr4	187522449	FAT1	G	A	481	326	0.678	p.R3872*		2.006	1.787
chr5	1295228	TERT	G	A	125	90	0.720	FIVE_PRIME_FLANK		1.910	1.830
chr7	6426850	RAC1	G	A	457	153	0.335	p.G15S		1.978	0.874
chr9	21971208	CDKN2A	C	T	314	238	0.758	SPLICE_SITE		1.999	1.994
chr9	139411802	NOTCH1	C	T	367	116	0.316	p.E493K	Uncertain	1.999	0.832
chr9	139413945	NOTCH1	C	G	345	122	0.354	p.C272S		1.999	0.930

b

Representative summary figures CNA-quiet oral cavity SCCs

Sample 1152

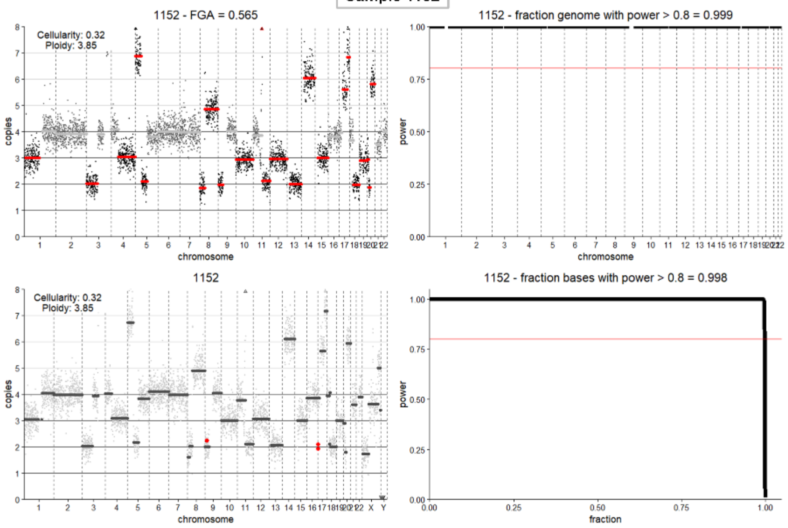

CHROM	POS	GENE	REF	ALT	DP	AD	VAF	change	ClinVar	Copynumbers	Mutant copies
chr17	7578231	TP53	CA	C	609	144	0.236	p.L206fs		3.864	1.919
chr17	7579345	TP53	CAAG	C	618	159	0.257	p.F113del		3.864	2.088
chr9	21971203	CDKN2A	AT	A	355	127	0.358	p.M52fs		2.012	2.240

Sample 704

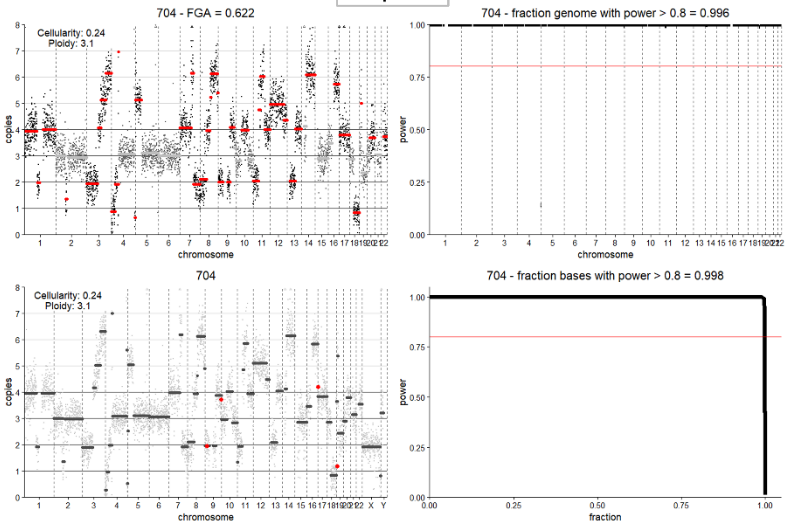

CHROM	POS	GENE	REF	ALT	DP	AD	VAF	change	ClinVar	Copynumbers	Mutant copies
chr17	7578203	TP53	C	T	715	295	0.413	p.V216M	Conflicting	3.834	4.195
chr19	10600395	KEAP1	G	A	753	75	0.100	p.A487V		5.384	1.167
chr9	21974807	CDKN2A	C	CT	377	88	0.233	p.S7fs		1.959	1.936
chr9	139401368	NOTCH1	CG	C	553	201	0.363	p.R1234fs		3.876	3.711

Supplementary Fig. 6 | Summary figures of somatic variants and called segments. a-b, Representative summary figures of **a,** copy number alteration (CNA)-quiet and **b,** CNA-other oral cavity squamous cell carcinomas (OCSCCs). Called segments in upper left panel in red, as output from low coverage whole genome sequencing (lcWGS). Segments are shown in grey and highlighted in red when called. In the lower left panel the somatic variant copies (in red) are plotted on the copy numbers. The table shows details on the variants. In the two right panels are the power to call segments (upper panel) and variants (lower panel). Samples were required to have > 80% power over at least 80% of the genome.

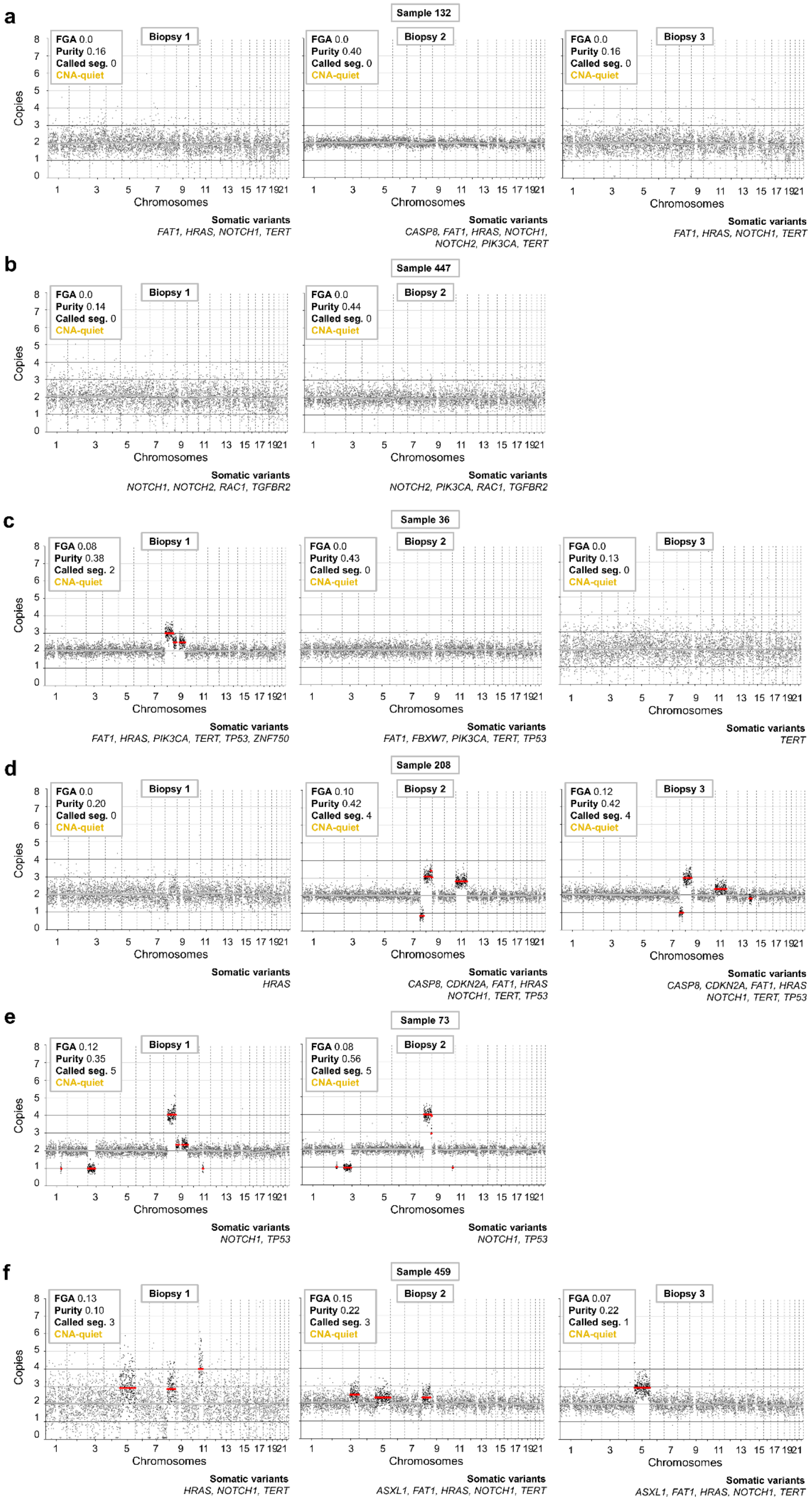
a
Sample 132
Biopsy 1
FGA 0.0
Purity 0.16
Called seg. 0
CNA-quiet
Copies
Chromosomes
Somatic variants
FAT1, HRAS, NOTCH1, TERT
Biopsy 2
FGA 0.0
Purity 0.40
Called seg. 0
CNA-quiet
Somatic variants
CASP8, FAT1, HRAS, NOTCH1, NOTCH2, PIK3CA, TERT
Biopsy 3
FGA 0.0
Purity 0.16
Called seg. 0
CNA-quiet
Somatic variants
FAT1, HRAS, NOTCH1, TERT
b
Sample 447
Biopsy 1
FGA 0.0
Purity 0.14
Called seg. 0
CNA-quiet
Somatic variants
NOTCH1, NOTCH2, RAC1, TGFBR2
Biopsy 2
FGA 0.0
Purity 0.44
Called seg. 0
CNA-quiet
Somatic variants
NOTCH2, PIK3CA, RAC1, TGFBR2
c
Sample 36
Biopsy 1
FGA 0.08
Purity 0.38
Called seg. 2
CNA-quiet
Somatic variants
FAT1, HRAS, PIK3CA, TERT, TP53, ZNF750
Biopsy 2
FGA 0.0
Purity 0.43
Called seg. 0
CNA-quiet
Somatic variants
FAT1, FBXW7, PIK3CA, TERT, TP53
Biopsy 3
FGA 0.0
Purity 0.13
Called seg. 0
CNA-quiet
Somatic variants
TERT
d
Sample 208
Biopsy 1
FGA 0.0
Purity 0.20
Called seg. 0
CNA-quiet
Somatic variants
HRAS
Biopsy 2
FGA 0.10
Purity 0.42
Called seg. 4
CNA-quiet
Somatic variants
CASP8, CDKN2A, FAT1, HRAS NOTCH1, TERT, TP53
Biopsy 3
FGA 0.12
Purity 0.42
Called seg. 4
CNA-quiet
Somatic variants
CASP8, CDKN2A, FAT1, HRAS NOTCH1, TERT, TP53
e
Sample 73
Biopsy 1
FGA 0.12
Purity 0.35
Called seg. 5
CNA-quiet
Somatic variants
NOTCH1, TP53
Biopsy 2
FGA 0.08
Purity 0.56
Called seg. 5
CNA-quiet
Somatic variants
NOTCH1, TP53
f
Sample 459
Biopsy 1
FGA 0.13
Purity 0.10
Called seg. 3
CNA-quiet
Somatic variants
HRAS, NOTCH1, TERT
Biopsy 2
FGA 0.15
Purity 0.22
Called seg. 3
CNA-quiet
Somatic variants
ASXL1, FAT1, HRAS, NOTCH1, TERT
Biopsy 3
FGA 0.07
Purity 0.22
Called seg. 1
CNA-quiet
Somatic variants
ASXL1, FAT1, HRAS, NOTCH1, TERT

Supplementary Fig. 7 | Multiple tumor core biopsies to test for copy number alteration (CNA) intratumor heterogeneity. a-f, Copy number plots of Sample **a,** 132, **b,** 447, **c,** 36, **d,** 208, **e,** 73 and **f,** 459. Segments are shown in grey and highlighted in red when called, as output from low coverage whole genome sequencing (lcWGS).

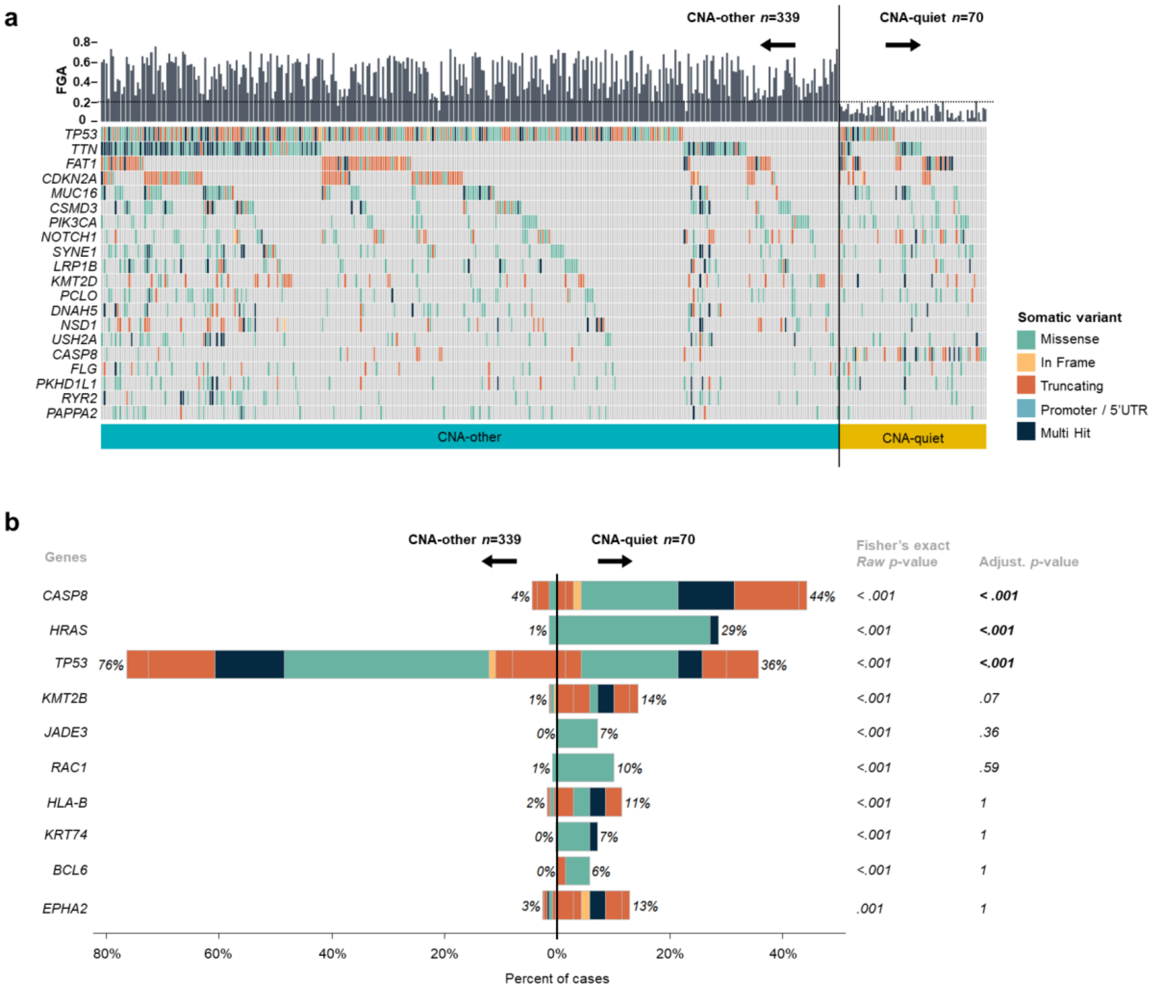

Supplementary Fig. 8 | Screening for differential mutations between 339 copy number alteration (CNA)-other versus 70 CNA-quiet head and neck squamous cell carcinomas (HNSCCs). Mutations of 15,881 genes in the 409 HPV-negative HNSCCs of the cancer genome atlas (TCGA) were analyzed. Results are listed in in Supplementary Table 7. **a,** Oncoplot of somatic variants of 20 most frequently mutated genes in 409 HPV-negative HNSCC samples. 339 CNA-other on the left (fraction genome altered (FGA) ≥ 0.20 and/or ≥ 20 called segments) and 70 CNA-quiet HNSCC on the right (FGA < 0.20 and < 20 called segments). Colors represent somatic variant classifications as listed in Supplementary Table 8. **b,** Fisher's exact test to compare gene mutation frequency between CNA-other and -quiet HNSCC. Raw as well as adjusted p-values (using Benjamini-Hochberg false discovery rate correction) are displayed; 10 genes with lowest raw p-values are displayed. Source data are provided with this paper.

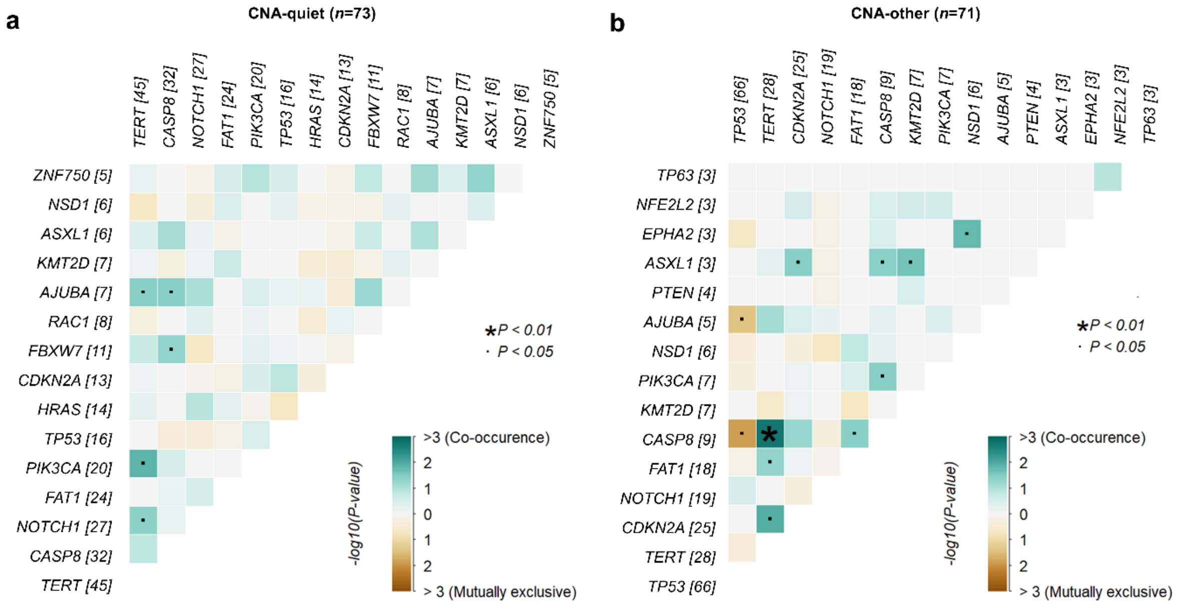

Supplementary Fig. 9 | Mutually exclusive and co-occurrence analysis. Pair-wise Fisher's Exact test to detect co-occurrence or mutually exclusive events with * $p < .01$ and • $p < .05$. in **a,** copy number alteration (CNA)-quiet (*n*=73) and **b,** CNA-other OCSCC (*n*=71). 15 most prevalent genes are displayed with mutation count between square brackets. Source data are provided with this paper.

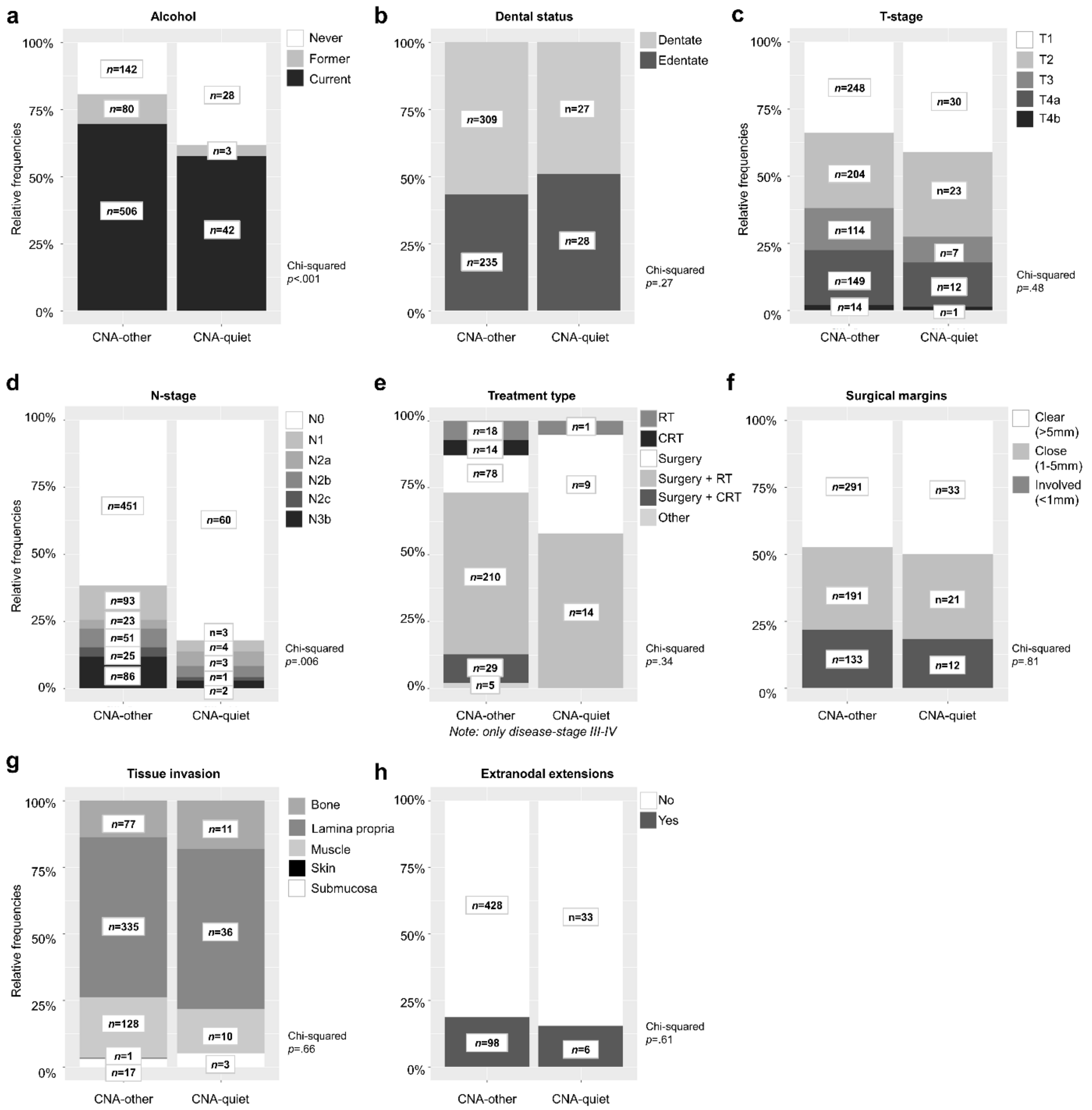

Supplementary Fig. 10 | Comparison between copy number alteration (CNA)-quiet and -other oral cavity SCC. a-g, Relative frequencies and number of patients are depicted in the bar graph per category of **a,** alcohol use **b,** dental care **c,** T-stage, **d,** N-stage, **e,** treatment type from advanced disease-staged (III-IV) treated with curative intent, **f,** surgical margins, **g,** tissue invasion and **h,** presence of extranodal extensions in CNA-quiet and -other groups. T-, and N-stage according to TNM classification 8th edition, 2017. Pathological stage was used when available; when the patient was not treated with surgery, clinical stage was used. *p*-values were obtained using a Chi-squared test. Unknowns were excluded from the analysis. Source data are provided with this paper.

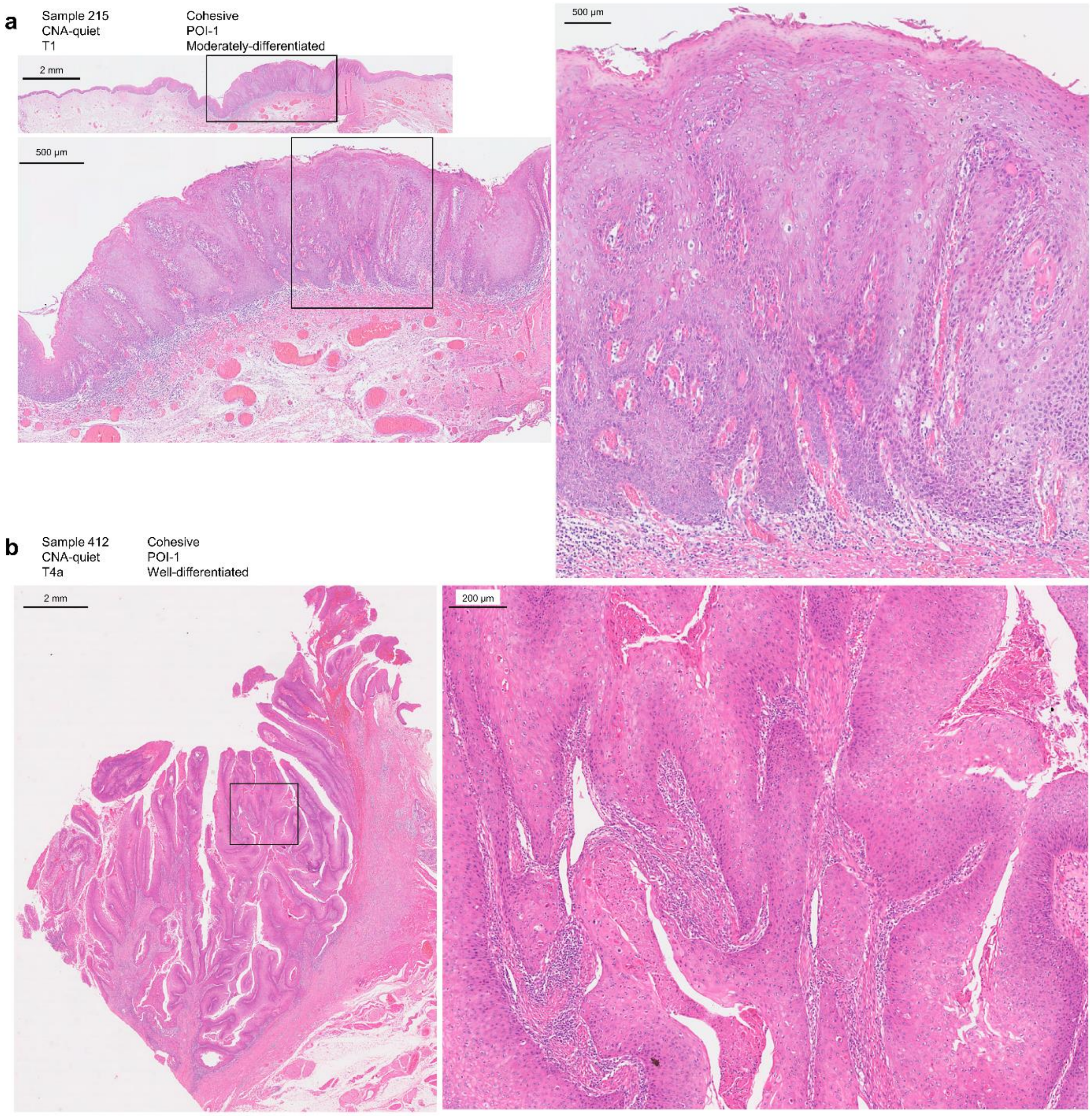

Supplementary Fig. 11 | Representative histopathology of copy number alteration (CNA)-quiet oral cavity squamous cell carcinoma (OCSCC). a, Sample 215: T1, cohesive, pattern of invasion (POI)-1, moderately-differentiated CNA-quiet OCSCC. **b,** Sample 412: T4a, cohesive, POI-1, well-differentiated CNA-quiet OCSCC. POI was scored by a trained pathologist as reported by Heerema *et al.* (4).

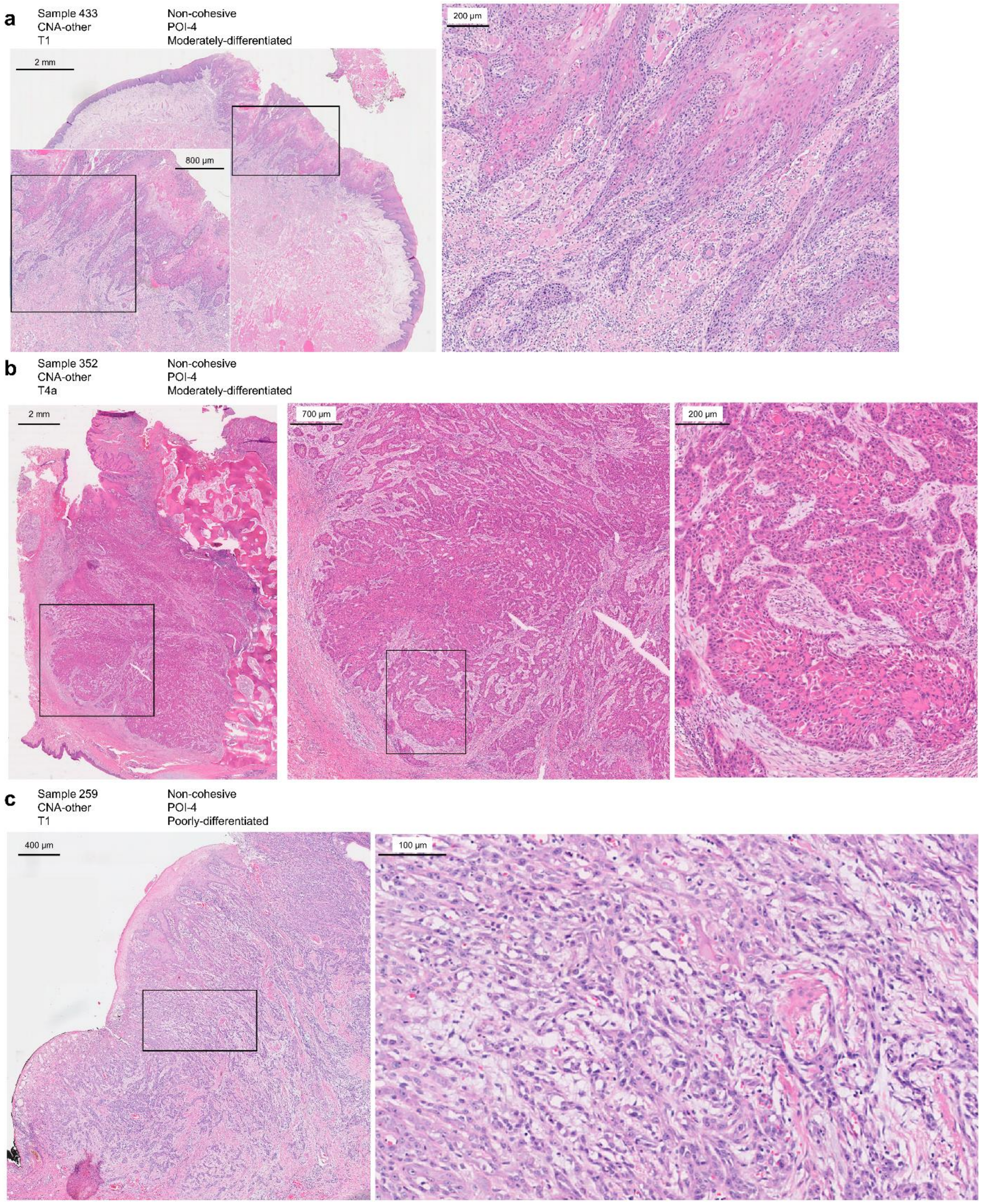

Supplementary Fig. 12 | Representative histopathology of copy number alteration (CNA)-other oral cavity squamous cell carcinoma (OCSCC). a, Sample 433: T1, non-cohesive, pattern of invasion (POI)-4, moderately-differentiated CNA-other OCSCC. **b,** Sample 352: T4a, non-cohesive, POI-4, moderately-differentiated CNA-other OCSCC. **c,** Sample 259: T1, non-cohesive, POI-4, poorly-differentiated CNA-other OCSCC. POI was scored by a trained pathologist as reported by Heerema *et al.* (4).

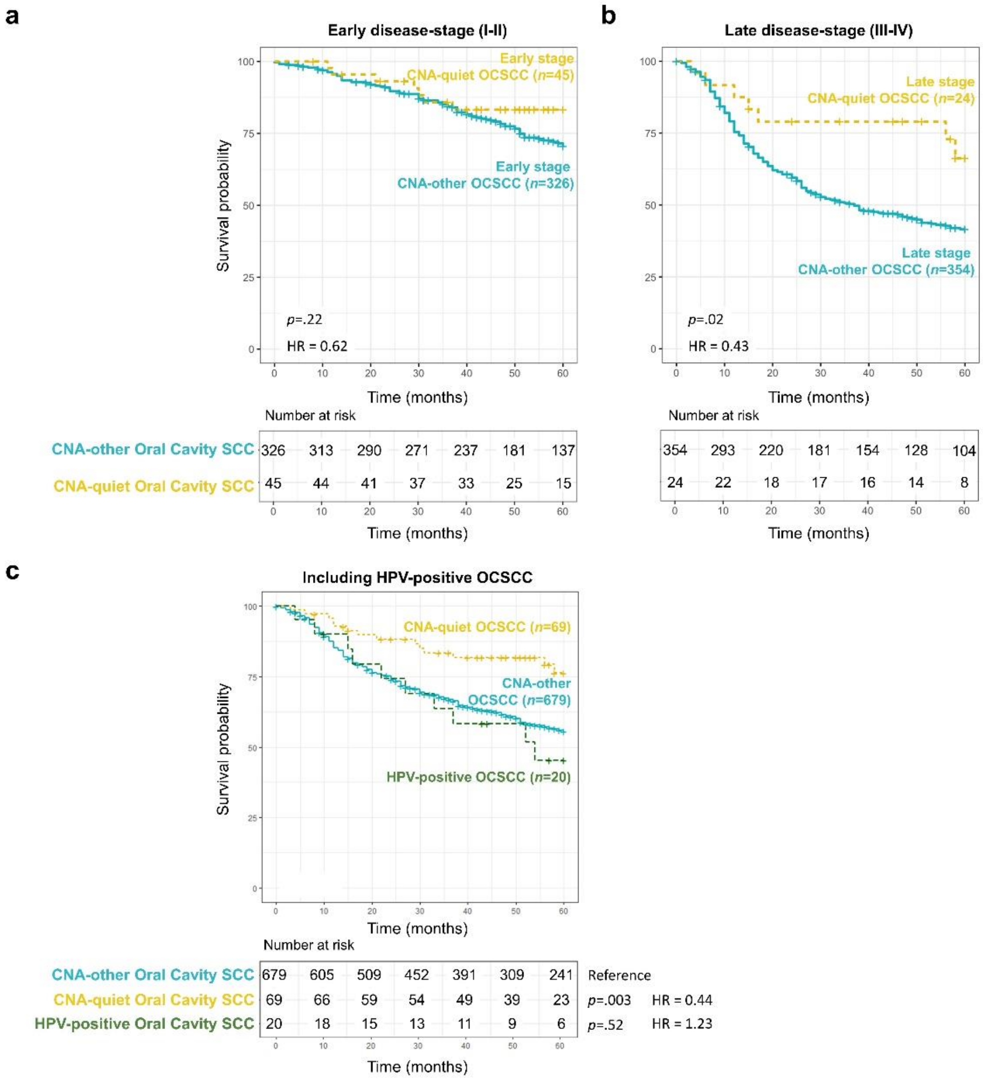

Supplementary Fig. 13 | Survival analyses. Kaplan-Meier curve for CNA-quiet and -other OCSCC with p-value displayed, obtained by log-rank test. Patients with curative treatment intent were included in the survival analysis; patients with palliative intent were excluded. Analysis performed for **a,** early disease-stage (I-II), **b,** late disease-stage (III-IV) and **c,** including human papilloma virus (HPV)-positive OCSCC. Source data are provided with this paper.

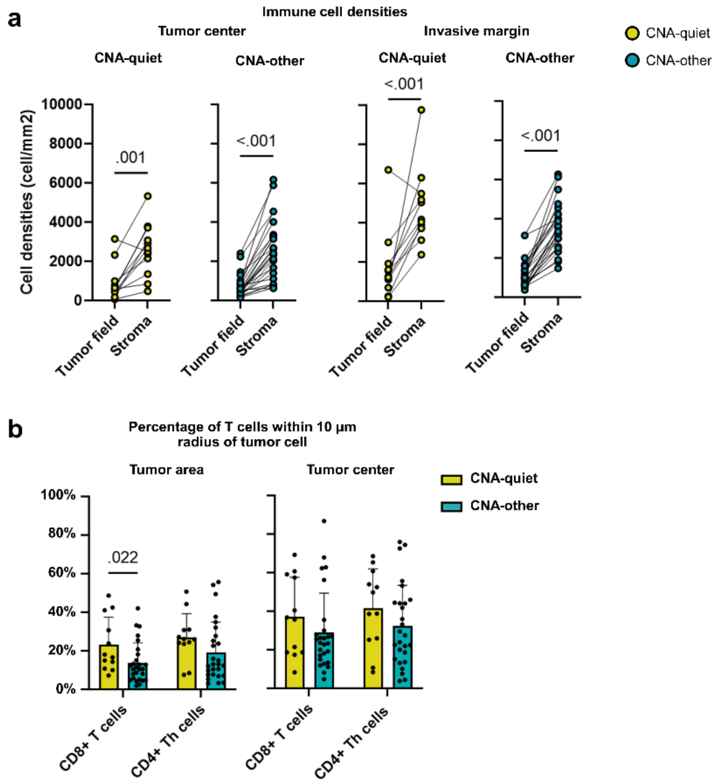

Supplementary Fig. 14 | Multiplex immunohistochemistry to characterize the tumor immune microenvironment of copy number alteration (CNA)-quiet (*n*=12) and -other (*n*=27) oral cavity squamous cell carcinomas. a, Total immune cell (T cells, B cells and CD163+ macrophages together) densities (in cells/mm2) in tumor field and stromal compartments within tumor center and invasive margin of CNA-quiet (yellow) and -other (blue) OCSCC. *p*-values were obtained by a two-tailed paired nonparametric Wilcoxon rank-sum test. **b,** Percentage of CD8+ T cells and CD4+ T helper cells within 10 µm radius of a tumor cell in the tumor area (left) and tumor center (right) of CNA-quiet and –other OCSCC. *p*-values were obtained by multiple unpaired non-parametric Mann-Whitney tests. Source data are provided with this paper. Data are presented as mean and error bars represent standard deviation.

References

1. Nulton TJ, Olex AL, Dozmorov M, Morgan IM, Windle B. Analysis of The Cancer Genome Atlas sequencing data reveals novel properties of the human papillomavirus 16 genome in head and neck squamous cell carcinoma. Oncotarget. 2017;8(11):17684-99.

2. Poell JB, Mendeville M, Sie D, Brink A, Brakenhoff RH, Ylstra B. ACE: absolute copy number estimation from low-coverage whole-genome sequencing data. Bioinformatics. 2019;35(16):2847-9.

3. Carter SL, Cibulskis K, Helman E, McKenna A, Shen H, Zack T, et al. Absolute quantification of somatic DNA alterations in human cancer. Nat Biotechnol. 2012;30(5):413-21.

4. Heerema MG, Melchers LJ, Roodenburg JL, Schuuring E, de Bock GH, van der Vegt B. Reproducibility and prognostic value of pattern of invasion scoring in low-stage oral squamous cell carcinoma. Histopathology. 2016;68(3):388-97.
