# Supplementary material for: Hallmarks of a genomically distinct subclass of head and neck cancer
Source: Nat Commun. 2024 Oct 20;15:9060. doi: 10.1038/s41467-024-53390-3 (PMC11491468; doi:10.1038/s41467-024-53390-3)
Supplement: Supplementary file 3 — Description of Additional Supplementary Files [file 41467_2024_53390_MOESM3_ESM.pdf]

Description of Additional Supplementary Files

Supplementary Data 1
Description: Patient and tumor characteristics of the HPV-negative HNSCC TCGA cohort with recalculated FGA

Supplementary Data 2
Description: Patient and tumor characteristics of the oral cavity SCC multicenter cohort

Supplementary Data 3
Description: List of somatic variants after targeted-enrichment sequencing of 73 CNA-quiet and 71 CNA-other cancers of the oral cavity SCC multicenter cohort

Supplementary Data 4
Description: Comparison of mutations CNA-quiet and CNA-other in the oral cavity SCC multicenter cohort

Supplementary Data 5
Description: List of *TP53* somatic variants in the oral cavity SCC multicenter cohort

Supplementary Data 6
Description: List of *PIK3CA* somatic variants in the oral cavity SCC multicenter cohort

Supplementary Data 7
Description: Screening for less frequently occurring mutations in HPV-negative TCGA cohort

Supplementary Data 8
Description: Variant classification for oncoplot and -bar

Supplementary Data 9
Description: multiplex immunohistochemistry antibodies and protocol steps for the manual Opal staining

Supplementary Data 10
Description: multiplex immunohistochemistry results of 12 CNA-quiet and 27 CNA-other oral cavity SCCs

Supplementary Data 11
Description: List of probes used for MLPA
