# Supplementary material for: Hallmarks of a genomically distinct subclass of head and neck cancer
Source: Nat Commun. 2024 Oct 20;15:9060. doi: 10.1038/s41467-024-53390-3 (PMC11491468; doi:10.1038/s41467-024-53390-3)
Supplement: Supplementary file 5 — Reporting Summary [file 41467_2024_53390_MOESM5_ESM.pdf]

nature portfolio

Corresponding author(s): Jos. B. Poell (j.poell@amsterdamumc.nl) and Ruud H. Brakenhoff (rh.brakenhoff@amsterdamumc.nl)

Last updated by author(s): Sep 25, 2024

Reporting Summary

Nature Portfolio wishes to improve the reproducibility of the work that we publish. This form provides structure for consistency and transparency in reporting. For further information on Nature Portfolio policies, see our Editorial Policies and the Editorial Policy Checklist.

Statistics

For all statistical analyses, confirm that the following items are present in the figure legend, table legend, main text, or Methods section.

n/a	Confirmed	
☐	☒	The exact sample size (*n*) for each experimental group/condition, given as a discrete number and unit of measurement
☐	☒	A statement on whether measurements were taken from distinct samples or whether the same sample was measured repeatedly
☐	☒	The statistical test(s) used AND whether they are one- or two-sided<br>*Only common tests should be described solely by name; describe more complex techniques in the Methods section.*
☐	☒	A description of all covariates tested
☐	☒	A description of any assumptions or corrections, such as tests of normality and adjustment for multiple comparisons
☐	☒	A full description of the statistical parameters including central tendency (e.g. means) or other basic estimates (e.g. regression coefficient) AND variation (e.g. standard deviation) or associated estimates of uncertainty (e.g. confidence intervals)
☐	☒	For null hypothesis testing, the test statistic (e.g. *F*, *t*, *r*) with confidence intervals, effect sizes, degrees of freedom and *P* value noted<br>*Give P values as exact values whenever suitable.*
☒	☐	For Bayesian analysis, information on the choice of priors and Markov chain Monte Carlo settings
☒	☐	For hierarchical and complex designs, identification of the appropriate level for tests and full reporting of outcomes
☐	☒	Estimates of effect sizes (e.g. Cohen's *d*, Pearson's *r*), indicating how they were calculated

Our web collection on statistics for biologists contains articles on many of the points above.

Software and code

Policy information about availability of computer code

Data collection	For acquisition of The Cancer Genome Atlas dataset, clinical (including ABSOLUTE estimates of tumor purity and ploidy) and segment data was downloaded from cBioPortal available via [https://www.cbioportal.org/study/summary?id=hnsc_tcga]. MAF files were acquired via the genomic data commons (GDC) portal availabe via [https://portal.gdc.cancer.gov/projects/TCGA-HNSC] using GDCquery_Maf of the TCGAbiolinks R package.<br>For data collection of the multiplex immunohistochemistry (mIHC) images the Vectra Polaris (PerkinElmer) was used.<br>Sequencing services were provided by the Genomics Facility of the Department of Clinical Genetics of Amsterdam UMC, entailing delivery of fastq and associated quality control files.
Data analysis	For data analysis of low coverage whole genome (lcWGS) analysis, Burrow-Wheeler Aligner (BWA) mem version 0.7.17 was used. For target-enrichment sequencing analysis, fgbio version 2.0.3 and GATK version 4.3.0.0 was used. In addition, for the Multiplex Ligation-dependent Probe Amplification (MLPA) analysis, Coffalyser.Net software version 140721.1958 (MRC Holland) was used. For the mIHC analysis, InForm version, 2.6 Qupath version 0.4.3 and GraphPad Prism version 9.3.1 were used.<br>Lastly, R version 4.2.1 was used. The following R packages were used throughout the manuscript: ACE version 1.18, HMMcopy version 1.42, QDNAseq version 1.36, DNAcopy version 3.17, VarScan version 2.4.4, Maftools version 2.14.0, survival version 3.5-5 and survminer version 0.4.9.<br>All software is freely available. Code and custom tools are available through FigShare (https://figshare.com/projects/Hallmarks_of_Copy_Number_Alteration-Quiet_Oral_Cavity_Tumors/193910).

For manuscripts utilizing custom algorithms or software that are central to the research but not yet described in published literature, software must be made available to editors and reviewers. We strongly encourage code deposition in a community repository (e.g. GitHub). See the Nature Portfolio guidelines for submitting code & software for further information.

Data

Policy information about availability of data

All manuscripts must include a data availability statement. This statement should provide the following information, where applicable:

- Accession codes, unique identifiers, or web links for publicly available datasets
- A description of any restrictions on data availability
- For clinical datasets or third party data, please ensure that the statement adheres to our policy

The raw sequencing data generated in this study have been deposited in the European Genome-phenome Archive (EGA) under accession number EGAD50000000790 via [https://ega-archive.org/datasets/EGAD50000000790]. Data will be made available under a data transfer agreement that will only contain statements on acknowledgement to the source publication in manuscripts using the data, commercial use of the data (not allowed), transfer of the data to other parties (not allowed), the aim of the study and the estimated time required for the planned analyses, in practice 1 to 3 years, but as long as needed. The mIHC imaging data generated in this study is deposited in the bioimage archive database and available under the accession number S-BIAD1352 via [https://www.ebi.ac.uk/biostudies/BioImages/studies/S-BIAD1352]. Publicly available clinical data of 530 HNSCC samples from TCGA were downloaded from cBioPortal and available via [https://www.cbioportal.org/study/clinicalData?id=hnsc_tcga]. Segment data derived from Affymetrix SNP 6.0 array were downloaded from cBioPortal available via [https://www.cbioportal.org/study/summary?id=hnsc_tcga]. MAF files were acquired via the genomic data commons (GDC) portal using GDCquery_Maf of the TCGAbiolinks R package. Purity and ploidy estimates of samples using ABSOLUTE were obtained from supplemental data from the 2018 Pan-Cancer Atlas publications, available through the GDC website via [https://portal.gdc.cancer.gov/projects/TCGA-HNSC]. Source data are provided with this paper.

Research involving human participants, their data, or biological material

Policy information about studies with human participants or human data. See also policy information about sex, gender (identity/presentation), and sexual orientation and race, ethnicity and racism.

Reporting on sex and gender	Sex was used in the current study, as reported in the patient file. Gender has not been documented or used in the study. Both, female and male sex were included. In the cancer genome atlas cohort (TCGA) consists of 125 female (31%) and 284 male (69%) (Supplementary Data 1) and the multicenter oral cavity squamous cell carcinoma (OCSCC) cohort consists of 379 female (42%) and 521 male (58%) (Supplementary Data 2).
Reporting on race, ethnicity, or other socially relevant groupings	Ethnicity or other socially relevant groupings were not reported for the Dutch OCSCC cohort.
Population characteristics	All patient characteristics are detailed in Supplementary Data 1 (TCGA cohort), Table 1 and Supplementary Data 2 (multicenter OCSCC cohort)
Recruitment	Unbiased, consecutive cohort of patients with an oral cavity tumor treated in either the Amsterdam UMC (Amsterdam, the Netherlands) or the Erasmus MC (Rotterdam, the Netherlands) hospital between 2008 and 2014 were recruited. Patients were excluded with an HPV DNA-positive tumor.
Ethics oversight	The study protocol for this retrospective study was approved by the Institutional Review Board (IRB) at Amsterdam UMC location VUmc under number 2021-0511. Signed informed consent was not required according to the IRB because of the retrospective nature of the study, the number of patients no longer alive, the notion that none of the patients objected to the secondary use of available clinical data and tissue samples for research, and pseudo-anonymization of all presented data. Privacy guidelines were followed according to the EU General Data Protection Regulation.

Note that full information on the approval of the study protocol must also be provided in the manuscript.

Field-specific reporting

Please select the one below that is the best fit for your research. If you are not sure, read the appropriate sections before making your selection.

☒ Life sciences ☐ Behavioural & social sciences ☐ Ecological, evolutionary & environmental sciences

For a reference copy of the document with all sections, see nature.com/documents/nr-reporting-summary-flat.pdf

Life sciences study design

All studies must disclose on these points even when the disclosure is negative.

Sample size	The sample size (listed in Supplementary Data 1, Table 1 and Supplementary Data 2) was determined based on data availability.
Data exclusions	Patients with an HPV DNA-positive tumor were excluded since this study focused on HPV-negative tumors.
Replication	Two independent cohorts were studied. The TCGA data was reproduced, and we studied a novel cohort from the Netherlands. For the DNA sequencing and multiplex immunohistochemistry (mIHC) analysis, no replication was performed.
Randomization	This is an observational study were randomization is not applicable.

Blinding	All analyses were performed blinded. In addition, the trained head and neck pathologist scoring for histological features such as differentiation grade, invasion pattern, and pattern of invasion, was not aware of the CNA classification.

Reporting for specific materials, systems and methods

We require information from authors about some types of materials, experimental systems and methods used in many studies. Here, indicate whether each material, system or method listed is relevant to your study. If you are not sure if a list item applies to your research, read the appropriate section before selecting a response.

Materials & experimental systems

n/a	Involved in the study	
☐	☒	Antibodies
☒	☐	Eukaryotic cell lines
☒	☐	Palaeontology and archaeology
☒	☐	Animals and other organisms
☐	☒	Clinical data
☒	☐	Dual use research of concern
☒	☐	Plants

Methods

n/a	Involved in the study	
☒	☐	ChIP-seq
☒	☐	Flow cytometry
☒	☐	MRI-based neuroimaging

Antibodies

Antibodies used

anti-CD44v6 (VUmc, clone U36, produced in-house, 1:100), anti-CD19 (Abcam, clone EPR5906, product# Ab134114, 1:100, lot# GR3278793-30), anti-CD3 (Dako, clone PAb, product# A0452, 1:100, lot# 20068606), anti-CD8 (Dako, clone C8/144B, product# M7103, 1:100, lot# 20071297), anti-FoxP3 (Abcam, clone 236A/E7, product# Ab20034, 1:100, lot# GR3438341-2), anti-CD163 (LEICA, clone 10D6, product# NCL-L-CD163, 1:25, lot# 6101868)

Validation

Anti-CD44v6 is produced in-house, following citations are available:
https://pubmed.ncbi.nlm.nih.gov/8364934/
https://onlinelibrary.wiley.com/doi/abs/10.1002/%28SICI%291097-0215%2819961115%2968%3A4%3C520%3A%3AAID-IJC19%3E3.0.CO%3B2-8?sid=nlm%3Apubmed
https://link.springer.com/article/10.1007/s002620050406
https://pubs.acs.org/doi/epdf/10.1021/acs.bioconjchem.2c00164

For all other antibodies, validation and citations are available in the company website:
Anti-CD19 https://www.abcam.com/en-nl/products/primary-antibodies/cd19-antibody-epr5906-ab134114#
Anti-CD3 https://www.agilent.com/en/product/immunohistochemistry/antibodies-controls/primary-antibodies/cd3-(concentrate)-76133
Anti-CD8 https://www.agilent.com/en/product/immunohistochemistry/antibodies-controls/primary-antibodies/cd8-(concentrate)-76631
Anti-FoxP3 https://www.abcam.com/en-nl/products/primary-antibodies/foxp3-antibody-236a-e7-ab20034
Anti-CD163 https://shop.leicabiosystems.com/ihc-ish/ihc-primary-antibodies/pid-cd163

In addition, as validation for the multiplex immunohistochemistry Opal panel, we performed the following steps:
- Single stains developed with DAB.
- Matched Opal to antibodies based on the brightness of Opal and the strength of antigen expression.
- Single stains with Opal were performed until normalized counts between 10-20 were reached for each single stain (with changesin dilution or antibody if necessary). These were also performed on consecutive slides using DAB staining for comparison.
- Antibody order was determined by trying each antibody in all possible positions.
- Fluoresence minus one (FMO) experiments were performed to check for sterical hindrance.

Clinical data

Policy information about clinical studies
All manuscripts should comply with the ICMJE guidelines for publication of clinical research and a completed CONSORT checklist must be included with all submissions.

Clinical trial registration	*Provide the trial registration number from ClinicalTrials.gov or an equivalent agency.*
Study protocol	*Note where the full trial protocol can be accessed OR if not available, explain why.*
Data collection	*Describe the settings and locales of data collection, noting the time periods of recruitment and data collection.*
Outcomes	*Describe how you pre-defined primary and secondary outcome measures and how you assessed these measures.*

Plants

Seed stocks

Report on the source of all seed stocks or other plant material used. If applicable, state the seed stock centre and catalogue number. If plant specimens were collected from the field, describe the collection location, date and sampling procedures.

Novel plant genotypes

Describe the methods by which all novel plant genotypes were produced. This includes those generated by transgenic approaches, gene editing, chemical/radiation-based mutagenesis and hybridization. For transgenic lines, describe the transformation method, the number of independent lines analyzed and the generation upon which experiments were performed. For gene-edited lines, describe the editor used, the endogenous sequence targeted for editing, the targeting guide RNA sequence (if applicable) and how the editor was applied.

Authentication

Describe any authentication procedures for each seed stock used or novel genotype generated. Describe any experiments used to assess the effect of a mutation and, where applicable, how potential secondary effects (e.g. second site T-DNA insertions, mosiacism, off-target gene editing) were examined.
